# Dental Caries Risk Studies Revisited: Causal Approaches Needed for Future Inquiries

**DOI:** 10.3390/ijerph6122992

**Published:** 2009-11-30

**Authors:** Jolanta Aleksejūnienė, Dorthe Holst, Vilma Brukienė

**Affiliations:** 1 Department of Oral Health Sciences, Faculty of Dentistry, The University of British Columbia, 2199 Wesbrook Mall, Vancouver, BC, V6T 1Z3, Canada; 2 Department of Community Dentistry, Faculty of Dentistry, University of Oslo, Blindern POB 1052, 0316 Oslo, Norway; E-Mail: dholst@odont.uio.no; 3 Institute of Odontology, Faculty of Medicine, Vilnius University, Žalgirio st. 115, Vilnius 08217, Lithuania; E-Mail: vilma.brukiene@mf.vu.lt

**Keywords:** caries risk, prediction, multi-risk, explanation, causal studies

## Abstract

Prediction of high-risk individuals and the multi-risk approach are common inquiries in caries risk epidemiology. These studies prepared the ground for future studies; specific hypotheses about causal patterns can now be formulated and tested applying advanced statistical methods designed for causal studies, such as structural equation modeling, path analysis and multilevel modeling. Causal studies should employ measurements, analyses and interpretation of findings, which are in accordance to causal aims. Examples of causal empirical studies from medical and oral research are presented.

## 1. Common Approaches in Caries Risk Studies

Two main approaches can be identified in caries risk studies: prediction of high-risk individuals and multi-risk assessment of disease patterns in populations. In the prediction approach, studies seek to identify characteristics of high-risk and low-risk individuals [1]. In the multi-risk approach, multiple factors are studied as risks related to dental caries. Multi-risk studies seek to identify risks or to explain variations in caries occurrence by evaluating a combined effect of multiple risk factors. Some caries risk studies combined the two aforementioned approaches. The main focus of both approaches is to find the strongest factors helping to identify either high risk individuals or risk factors for dental caries.

## 2. Literature Search for Caries Risk Studies

The present work does not intend to comprehensively review all studies within the area of caries risk epidemiology, but rather aims to discuss present approaches and their methodology as tools for further advancement of the field. However, to form a backdrop for this project, the MEDLINE (1950 to Present with daily update) database was searched electronically for the MeSH Subject Headings “Dental caries” and “Epidemiological studies” and for the truncated keywords: “caus*”, “predict*”, “expla*”, and “risk*”. The search history is presented in Table 1. A total of 768 references were extracted and subsequently abstracts and reference lists were manually checked to identify studies with a focus on dental caries risk in populations. A total of 222 references were used to analyze current approaches in caries risk studies.

An overview suggests that there is a general lack of consistency in reports from the literature and no clear distinction can be made between studies with different aims. Notions such as ‘risk factors’, ‘risk indicators’, ‘predictors’ and ‘explanatory factors’ are frequently used interchangeably [2,3], making it difficult to interpret existing literature. In the present work, the studies which mainly sought to find characteristics of high-risk individuals were noted as ‘prediction studies’ and the studies which sought to find risk factors for caries were attributed to the ‘multi-risk studies’. This review classified studies into two main approaches: a prediction approach and a multi-risk approach.

## 3. Prediction Studies

Prediction studies tested many factors for their ability to predict high- and low-risk individuals and many different factors were evaluated as predictors for caries. Sensitivity, specificity, positive and/or negative predictive values are usually reported in these studies, indicating the success of prediction [4–6].

The prediction models usually presented varying ranges of sensitivity (29%–70%) [7–12] and specificity (65–80%) [13,14]. Different measures of social factors and past caries experience have been shown as the best predictors of high risk groups [15], while other factors usually added little to the accuracy of the prediction [14,16,17]. Opinions differ on the value of past caries experience as a predictor of future caries. Critics argue that one should aim at predicting disease occurrence before there are signs of past disease experience [18]. Other problems related to the inclusion of past caries related measurements into multiple regression models will be discussed later.

Averaged individual risk measures such as the odds ratio (OR) and the relative risk ratio (RR) are usually presented as measures of the size of an effect in prediction studies [19]. In dental research, the confidence intervals for OR are usually wide. A few examples may illustrate this point. Daily consumption of sweets at the age of 3 years (OR 2.7; 95% CI 1.5–4.8) was associated with a caries increment between 7 and 10 years of age [20]. Another study reported that, after controlling for fluoride history, medical problems, diet, and self-reported oral hygiene, children with attention-deficit hyperactivity disorder (ADHD) had nearly 12 times the odds (OR = 11.98; 95% CI 1.13, 91.81) of having a high DMFT score than children who did not have ADHD [21]. It is also important to emphasize that if the OR is interpreted inappropriately *i.e.*, as RR, it will always overstate the effect size [22].

## 4. Multi-Risk Studies

Similar to prediction studies, numerous multi-risk studies have been reported. In these studies, caries has been associated with multiple factors [23–27]. This approach has enabled researchers to detect the effects of single risk factors nestled in a background of multiple risk factors [28]. However, a rather common approach in these studies is that the risk for disease is usually assumed as residing within individuals and their personal behavior. Regression coefficients are usual risk measures in multi-risk studies. It is important to consider that risk ratios or regression coefficients are only relative measures of risk. For example, weights of regression coefficients are highly unstable because when a variable is added to or subtracted from the regression equation, the weights of regression coefficients change. Moreover, the values of regression coefficients tend also to fluctuate considerably from study to study and from sample to sample [29], *i.e.*, the same risk factors are presented with different risk ratios or regression coefficients in different studies. These inconsistent findings should not be surprising as the regression analysis adjusts for the effects of all the measured factors in any particular study; thus resulting estimates such as risk ratios, odds ratios or regression coefficients are adjusted effects. After reviewing these studies, in which different sets of independent variables were analyzed it is still unclear whether differences in the effects of risk factors should be attributed to individual differences, to differences inherent in different populations, to statistical analysis; or to all of these. This makes generalization of the findings difficult.

Similar to prediction studies, different measures of past caries experience are often included in analytical models of multi-risk studies. It is important to emphasize that although inclusion of measures related to past caries experience can be justified in prediction studies, this should be discouraged in multi-risk studies. For example, an inclusion of variables, such as ‘the number of decayed teeth’ [30,31] or some other indicators of ‘past caries experience’ [32,33], and relating them to caries in a multi-risk study is inappropriate because there are consequences of including past caries experience measures for both prediction and multi-risk models. The main problem with inclusion of past caries measures into analyses is that they will hide the effects of weaker, although as important, indicators of high risk individuals or, alternatively, of other caries risks. This occurs due to the fact that strong predictors (past caries experience measures) are used as indicators of the same progressing disease which they intend to predict or explain. Clinically, this could be interpreted as “past caries is a good predictor or a good explanatory factor of future caries”. From an analytical standpoint, this means that past caries measures are simultaneously introduced into both ends of the equation, namely in the independent set and in the dependent outcome. Unsurprisingly, analyses will identify them as the strongest contributors to both prediction and explanation. A few examples can illustrate this point; prediction studies [34,35] found factors related to past caries to be best predictors of individuals who will experience caries in the future and, alternatively, multirisk caries studies found past caries as the best explanatory factor for future caries [36,37]. Given that past caries measures are used for predicting high risk individuals, one still has to evaluate the effects of other important predictors. This can be done by supplemental analysis where past caries experience measures are excluded in order to enable the weaker predictors to show their contributory effects to future caries prediction.

## 5. Differences and Commonalities between Prediction and Multi-Risk Approaches

Different types of questions (aims) require different types of study design; consequently implications of findings from various approaches differ. We believe that this has not been emphasized enough in caries risk epidemiology.

**Figure 1.**
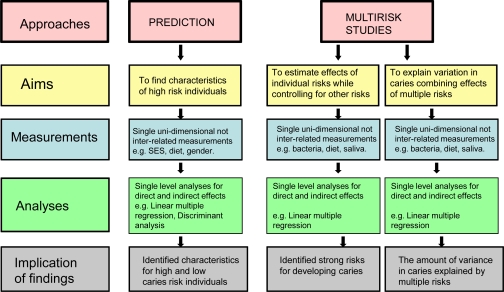
Current status of common caries risk epidemiology.

Figure 1 illustrates commonalities and differences between the two approaches as employed in the current caries risk epidemiology. Firstly, ‘prediction’ and ‘multi-risk’ approaches have different aims, but similar measurements and analyses have been chosen. Secondly, in both approaches the central focus is on individuals, *i.e.*, all risk measurements are tailored towards individual risk. In this way, group (sub-population, population) related risks are neither identified, nor assessed. Thirdly, in both approaches uni-dimensional (single aspect related) instead of multidimensional (comprehensive including multiple indicators) measurements for complex constructs are chosen. Fourthly, single level analyses are usually employed in both approaches, where the sequence of direct and indirect influences and interaction patterns are ignored. This means that concepts (measurements) of distant (e.g., socio-economic status), intermediate (e.g., toothbrushing frequency) as well as proximal biological influence (e.g., bacteriological load) will be treated equally as direct effects and dependency between them is not considered in multiple regression (MR) analyses, which are the most frequently employed statistical analysis in caries risk epidemiology.

It is important to remember that in MR models an assumption for independency has to be fulfilled; *i.e.*, factors to be tested as ‘predictors’ or ‘risk factors’ have to be independent and not interrelated if they are to be introduced simultaneously into MR models. Multicollinearity is a serious problem when study variables are interdependent, and it undermines the validity of regression coefficients. For example, the simultaneous testing of dental plaque scores and the number of bacteria per ml of plaque is a serious problem in MR models, which can give rise to spurious results. Despite the seriousness of the multicollinearity problem, it has been frequently overlooked in past dental research [38].

Based on the main differences in the two approaches, we suggest that interpretation of the findings in prediction and multi-risk studies as well as their implication should be different. For example, a common finding in prediction and multi-risk studies is that biological factors (proximal effects) show stronger associations than lifestyle (intermediate effects) or social factors (distal effects) in regards to predicting future caries activity. The reason for this can be partly attributed to analyses such as linear multiple regression where all factors are evaluated as equally direct effects. According to Victora *et al.* [39], when distal, intermediate and proximate factors are simultaneously included in a regression model, a reduction or elimination of the distal factors’ effects is observed. This observation may, frequently, lead researchers to think that proximal factors (usually biological) are more important than distal factors (usually indicators of socioeconomic position). Another substantial problem in multirisk caries studies is that the presence of site-specific risks, e.g., difference in risks even within the same oral cavity, is not accounted for in the present approaches. Obviously, this difficulty adds to the complexity of studying multirisks. Since different sites in the oral cavity present different risks, the susceptibility to these risks differs among individuals, and the susceptibility to dental caries also differs among population groups as well as among countries. We suggest that when looking at the development of chronic diseases, a comprehensive study of multirisk should be undertaken in which the operation of the risks at different levels is assessed. For example, four different levels (hierarchies) of operating factors can be identified in caries development even within the same country, namely site, tooth, individual and population group (sub-population) related factors. In future explanatory studies the interactions among factors across and within levels should also be modeled.

## 6. Limitations of Current Approaches

The main limitations of current caries risk epidemiology may be outlined as follows:

- Lack of success to predict high-risk individuals.
- Lack of success to explain variations in caries among individuals, population subgroups or between populations.
- Evidence for dental health promotion is insufficient; individual-based caries risk studies are plenty, while population-based risk studies are scarce.
- Current approaches cannot efficiently support population-based dental health preventive programs.

### Lack of success to predict high-risk individuals

It is important to consider that despite numerous attempts to predict disease, prediction models are inaccurate for targeting high-risk people as a large percentage of people truly at high risk would be missed by existing prediction models [40]. Moreover, the fact that prediction studies aiming to find high-risk individuals report higher specificity scores, *i.e.*, the ability to predict the individuals who will not develop disease, than sensitivity scores, *i.e.*, the ability to predict the individuals who will develop disease, speaks to the problem of disease prediction. It has been reported that changes in caries experience occurred throughout populations and are not confined to subgroups. Therefore, strategies limited to individuals ‘at risk’ would fail to deal with the majority of new caries lesions [41]. Moreover, doubt has been raised that no universally applicable multi-parameter predictive model is ever likely to be discovered [42], and that an accurate caries risk prediction model for use across all populations does not exist and might be unattainable [42,43].

### Lack of success to explain variations in caries among individuals, population subgroups or between populations

From the literature, it would appear that multi-risk studies are less helpful in explaining disease occurrence than prediction studies which try to predict high risk individuals because multi-risk studies at best explain only 50% of the variation of caries [19,44–47]. For example, a hierarchical logistic regression model explained only 15% of variation in self-perceived oral health among adolescents by means of four groups of independent variables, namely ‘socio-demographic’, ‘oral health behaviors’, ‘clinical oral health indicators’ and ‘subjective measures of oral health’ [48].

The multi-risk studies seek to find multiple risks for developing a disease. Greenland, a well-known scholar in epidemiology, describes “risk-factor epidemiology” as epidemiologic studies in which data are collected without any test–hypothesis being stipulated in advance, or in which data collected for other purposes are analyzed to look for associations among certain exposures and diseases [49].

From what does exist, the main limitation of multi-risk studies is due to problems with the study design, *i.e.*, although studies aimed to identify multiple risk factors for a disease or sought to explain a disease with multi-risk models, the measurements and analyses employed were in accordance with the aims of prediction studies, but not with the aims of multi-risk (explanatory studies). Moreover, the multi-risk approach is a deterministic approach to causation and has been suggested to be an outdated principle [50]. Furthermore, multi-risk (multi-association) models can be criticized because factors from different levels e.g., biological, lifestyle, or social, are treated as equally direct effects.

### Evidence for dental health promotion is insufficient: individual-based caries risk studies are plenty, while population-based risk studies are scarce

Dental researchers are currently focused on individuals without a proper consideration of risks inherent in population subgroups or populations. In providing evidence for the promotion of dental health, it is important to consider two distinctly different strategies, namely the ‘high-risk strategy’ and the ‘population strategy’. The individual focus in caries risk studies provides evidence for the ‘high-risk prevention strategy’ while population-based caries risk studies are necessary for the ‘population strategy’. These two distinctly different strategies have been suggested and discussed by Geoffrey Rose, an eminent epidemiologist, whose ideas transformed strategies for improving general health [51]. The combination of two strategies should be useful for reducing inequalities in dental health but their implementation should be approached differently.

At present, most evidence has been accumulated for the ‘high risk strategy’ which is adequate for dental practitioners focusing on individuals with the highest risk for caries. The main strength of this strategy is that prevention is matched individually; this increases the likelihood of a cost-effective use of resources. Concomitantly, the main weaknesses of the high-risk strategy are that successes may be palliative (temporary) and that the overall reduction of risks in a population may be small [52].

The ‘population strategy’ may provide long term results of maintaining good dental health as compared to the short term improvement gained from the ‘high risk strategy’, which targets only individuals at high risk for caries. The ‘population strategy’ may also be culturally appropriate and achieve a sustainable general change in the behavioral norms of socially conditioned behaviors [53]. The main limitations of the ‘population strategy’ are that it offers only small individual benefits and requires major societal shifts to achieve long term behavioral improvements [53].

### Current approaches cannot efficiently support population-based dental health preventive programs

The purpose of epidemiology is to acquire evidence regarding the patterns of a disease and its associated determinants, causes or risk factors and to apply that knowledge to improve public health [54,55]. This means population-focused prevention based on evidence is a cornerstone of public health [56].

Given that identification of causal pathways for oral health inequalities is essential for public health programs and policies [57], current approaches focusing on individual risks to caries have little to offer for public health-based health promotion strategies. The latter need evidence from studies where population (or subpopulation) related risks and pathways leading to a disease’s development are identified and adequately tested.

If only one or two factors were strongly associated with caries experience, it would be much easier to establish an effective and simple preventive system [58]. Given the multifactorial nature of caries development, current approaches of caries risk studies and their limitations do not offer sufficient evidence for planning interventions for population-based programs. Consequently, society cannot manage prevention effectively when variability, uncertainty, and limited causal knowledge characterizes decision making. It has been noted that population-level prevention and intervention must be re-examined in light of the limits of risk-factor findings at the individual level [59]. Consequently, an alternative approach is needed, in which patterns leading to disease occurrence are studied. Moreover, findings from studies about causes may provide crucial clues to the design of preventive interventions [60].

## 7. Towards Further Understanding of the Development of Dental Caries in Populations: A Causal Approach as an Alternative to Prediction and Multi-Risk Assessment

As things stand, it is not surprising that caries epidemiologists should feel increasingly frustrated. Like all sciences, epidemiology seeks to explain the causes of things [61]. Consequently, causation has been accepted to be an essential concept in epidemiology [62]. The longitudinal prospective design permits investigation of causes and outcomes [63]. However, the common way epidemiologists search for causes continues to be through the test of risk factors (potential causes), one-by-one even if these risk factors are each part of a multi-causal complex [64].

Towards a further understanding of causes or risks leading to the occurrence of disease or to the understanding of causes contributing to health maintenance in groups of individuals or populations, an alternative causally (explanation) oriented approach is needed. The expected implication goal of this approach is to identify and, if possible, influence the underlying causes of our society’s major health problems [65]. In this approach, the hierarchical structure of occurring events is acknowledged and causes are estimated at multiple levels of organization and within the context of both societies and individuals. Consequently, we believe that causal thinking should proceed with a deepened understanding of study design [66].

In the preliminary stages, it is important to decide the scope of the causal study, what is the topic of interest? how many levels/types of occurrences will be included to convey the necessary inter-relations? and which part of the causal web will be estimated? [67]. Subsequently, causal hypotheses should be developed to explain a particular phenomenon in which causal aims guide the choice of measurements, the analytical procedures and the interpretation of the results [68]. It is important to emphasize that the components of any causal pattern are themselves rich structures, where each part could be expanded to show its complex contents [69]. This means the different components of a study design should be considered thoroughly, *i.e.*, complex concepts must be measured multidimensionally, and analyses must have the ability to assess patterns where occurrences are multiple and interactive at different levels, e.g., biological, lifestyle and social.

## 8. Causal Studies—Focus on Measurements and Analyses

Limitations of the current design for studying complex patterns of oral disease may be mainly attributed to the choice of measurements and analyses. Thus, a different focus on measurements and different analyses are needed to facilitate the alternative approach in caries epidemiology.

### Re: Selection of measurements

Recent publications have criticized the weak conceptual foundation of both health status measures and the uses to which they are put [70]. Thus, the primary challenge for health researchers are conceptual considerations for measurements including (*a*) definition of health outcome; (*b*) determination of specific health or risk constructs relevant to the study’s objectives; and (*c*) specification of associations and patterns for hypothesis testing. Candidate measures need to be evaluated for how well they correspond to both the *a priori–*specified conceptual and methodological needs [70].

Regarding measurements, at least a few aspects should be considered; the most appropriate for the particular purpose, the benefits of multidimensional measurements and the scale on which the measurements will be taken.

Multidimensional indices should be used for measures related to population health, *i.e.*, measurements should consider more than one aspect of complex- structures [69] because of the unavoidable shortcoming of using a single measurement (unidimensional) as an adequate indicator of a complex (multidimensional) phenomenon [71]. This means that each complex concept should be indicated by a few highly interrelated measurements and many variables (measurements) may be needed to represent sequences of events at each of the chosen levels [72].

In dental caries research, any risk-related construct should be seen as a continuum, *i.e.*, from no risk to excessive risk exposure. Consequently, researchers with causal or explanatory aims should attempt, where possible to measure risks on interval scales in order to obtain accurate measurements [73,74]. This requirement is important because it has been reported that measuring inherently continuous phenomena with ordinal scales instead of using interval scales weakens the power to detect effects [75,76].

The integration of new approaches and new measurement tools demands methodological innovation and must include development of new concepts and related statistical models to help us understand complicated patterns of relationships among different concepts [77].

### Are prediction analyses suitable for causal inquiries?

Several statistical textbooks have suggested or recommended the use of multiple regression (LMR) for both prediction and explanation [78,79]. Therefore, not surprisingly, linear or logistic multiple regression (LMR) has been frequently employed in both prediction and multi-risk studies.

Regarding the analyses, prediction analyses cannot be chosen for testing models comprising interrelated measurements, as the main assumption for independence will obviously be violated. This means that analyses, such as simple linear multiple regression, currently employed in caries risk epidemiology have strong limitations when testing patterns of interaction among complex interrelated measurements. Moreover, currently employed analyses do not recognize that causal events may connect individuals, *i.e.*, the outcome in one individual is erroneously assumed to be independent of the outcome in other individuals [80]. However, this dependency among individuals or groups of individuals should be considered and its assessment approached in the study design.

It is important to emphasize that linear multiple regression has strong limitations for testing causal hypotheses or theory supported patterns. LMR analyses are not in accordance with explanatory or causal aims because strong factors hide the effects of weaker factors, interactions are not considered, and factors with both direct and indirect effects are treated equally. One of the possible reasons for the inadequacy of this analysis for causal testing is that LMR analysis was primarily designed for prediction studies aiming to find a few, not related and not necessarily causal indicators of high-risk individuals. A detailed discussion of limitations of LMR for testing causal patterns has been discussed elsewhere [81].

### Causal analyses are necessary for causal inquiries

It is important to consider the specific features of dental population-based research. Data on caries are usually collected with the tooth surface or the tooth as the unit of measurement, but subsequently data is analyzed by aggregating information at the level of the individual. It has been demonstrated that the precision of the estimates increased considerably when the tooth as compared to the individual was used as the unit of analysis [82].

The growing knowledge about multilevel interactions raises a question why so much health research continues to focus on single effects of specific factors, rather than elaborating the contextual nature of causal influences [83]. In order to estimate the precise extent of the relation between specific factors and the occurrence of oral disease, a coherent disease model is required. This model should also permit multivariate causal analysis to control for confounders and interactions. Only with such a disease model will it be possible to investigate causes of oral disease development in populations [84]. As mentioned earlier, studies employing causal methodologies or causal analyses are uncommon in dental epidemiology. Consequently, new statistical analyses should be sought.

Complex statistical methods are available which challenge researchers to test together interrelated multidimensional structures defined at different levels, and thus enable us to explore and assess more sophisticated models [85]. A few causal analyses have been overviewed by Greenland and Brumback [86]. A statistical tool such as structural equation modelling (SEM) or path analysis can be useful for testing causal hypotheses in caries risk epidemiology. The advantage of these analyses is that they require *a priori* hypothesized model which can be tested in a simultaneous analysis of the entire system of variables to determine the extent to which it is consistent with the data [87]. Compared with standard regression methods, the SEM or path analysis (PA) is guided by a theory or hypothesis driven approach, thus the resulting equations are a more accurate representation of the true causes of variation in the outcome variable [88]. By demanding that the pattern of intervariable relations be specified *a priori*, SEM and PA lend themselves well to the analysis of data for inferential purposes. By contrast, most other multivariate procedures are essentially descriptive in nature, so that the hypothesis testing is difficult, if not impossible [87].

In the social, medical and biological sciences, multilevel or hierarchically structured data are common [89]. Obviously, current approaches in caries risk epidemiology lack a systematic approach to correct for the clustering of dental disease within mouths. This could be addressed by using Multilevel Modeling statistical techniques, which accommodate the hierarchical structure of information [90]. These points were made clearly by oral epidemiologists, Newton and Bower, who presented new approaches to conceptualize and research causal networks [90]. A few applications of advanced statistical techniques will be presented in later sections.

Another advantage of advanced statistical techniques is that revisions of the models may be performed and subsequently tested. Moreover, the findings may give some clues for future inquiries or where to expand the present models in order to include missing links, *i.e.*, evidence can be acquired and built incrementally through a series of consecutive studies. For example, if an association between the two factors is known but is not strong, one can further hypothesize and subsequently test the missing links between the two factors. This way, evidence about chronic disease development can be built incrementally by continuously including newly identified causal (or risk related) links.

### Different treatment of ‘confounder’ variables in prediction and causal analyses

Consideration of confounding is fundamental to the design, analysis, and interpretation of studies intended to estimate causal effects [91]. In causal analyses, confounders will be treated differently than in the most frequently used analyses of current epidemiology. In current caries research, the usual treatment of confounders is to control for them and present magnitudes of risk effects for each factor after such control was employed. We would suggest that this approach should not be encouraged in causal studies because it limits the amount of acquired knowledge. For example, knowing that a risk factor influences two genders differently and controlling for it in multivariate analyses helps little to understand how or why these differences evolved. In causal analyses, causal patterns can be compared across gender groups, social classes, ethnic groups, or between groups residing in different geographic locations (e.g., low-fluoride *vs*. optimal fluoride). This feature of causal analyses allows us to identify similarities as well as differences within patterns, thus enhancing our causal understanding.

## 9. Empirical Examples of Causal Studies

A few examples from medical and from dental epidemiology were chosen to illustrate the evaluation of causal hypotheses.

### A medical example of a causal study

The purpose of this causal study [92] was to evaluate the causal relationships among arteriosclerotic risk factors, including age, smoking, alcoholic consumption, exercise, hypercholesterolemia, hypertriglyceridemia, and hypertension. The study hypothesis was that obesity leads to hyperlipidemia and the latter to hypertension. The extrinsic variables were age, smoking, alcohol consumption and exercise and the intrinsic variables: obesity, hyperlipidemia, and hypertension. The causal analysis was employed to test a hypothetical causal model applying the Structural Equation Modeling. Results showed that risk factors were directly and indirectly interrelated, and lifestyle variables (smoking, alcohol consumption, and exercise) influence almost all arteriosclerotic risk factors. The authors interpreted their findings and suggestions in a causal way, *“alcohol use increased the tendency toward obesity and then hyperlipidemia indirectly”*. Based on their findings, the authors make suggestions for a health prevention program that intends to modulate risk factors to prevent hyperlipidemia.

### Causal studies in dentistry

The study by Litt *et al.* applied a causally guided study design to investigate dental caries development in low-income children. Relationships among biological, cognitive, behavioral, and social variables were hypothesized [93]. All measurements were pre-tested on pilot studies (development of the measurement model). Subsequently, causal analyses (structural equation modeling) were employed to test the series of causal hypotheses and for the stepwise construction of the final model. Causal hypotheses modeled both direct and indirect effects and their patterns of interactions, e.g., ‘brushing and use of a baby bottle directly influence the number of *Streptoccoci mutans*, while sugar use directly influences numbers of *Streptoccoci mutans* and ‘past caries experience’ and ‘future caries experience’ is directly influenced by numbers of *Streptoccoci mutans* and past caries experience’. The final causal model presented paths of coefficients and their directionality to future caries experience. The goodness of fit of this model was very high as 99% of the covariance between the introduced factors was explained by the model. The likeliness of the model was also good (P = 0.47). The authors make their suggestions for preventive programs based on their findings. An interested reader can refer to a few other causal empirical studies [94–96].

A recent study by Donaldson *et al.* explored the effects of social class and dental attendance on oral health and concluded that the association between socioeconomic status (SES) and dental health (number of sound teeth) is partially explained by the pathway SES → barriers to dental attendance → dental attendance → number of sound teeth. Based on their findings, the authors suggest for future studies to explore other oral health related outcomes [97].

Bower *et al.* evaluated the effects of area deprivation on oral health applying multilevel modeling [98]. Interestingly, the authors discuss the limitation of their unidimensional indicator of area deprivation *“it is very difficult to capture essence of deprivation in one score”* and subsequently suggest using a multidimensional measure, ‘the Scottish Index of Multiple Deprivation 2004’, which consists of 31 indicators in the six domains of income, employment, housing, health, education, skills and training and geographic access to services [98].

## 10. Conclusions

The current approaches of caries risk epidemiology have limitations for further advancement of the science. Thus an alternative causal approach is proposed. This approach encourages thinking about causes at multiple levels of organization and within the context of both societies and individuals. The proposed approach is not in contradiction with current oral epidemiology and aims to preserve and build on the contributions of past eras.

## Figures and Tables

**Table 1.** The MEDLINE (1950 to Present with daily update) database search for dental caries risk studies in populations.

Steps	MeSH subject heading or keyword	Number of references	Search mode
1	exp Epidemiologic Studies	1,180,888	Advanced
2	dental caries.sh. not restor*.af. not endo*.af. not perio.af. not implant*.af.	26,910	Advanced
3	1 and 2	2,269	Advanced
4	predict*.mp. [mp = title, original title, abstract, name of substance word, subject heading word]	618,379	Advanced
5	expla*.mp. [mp = title, original title, abstract, name of substance word, subject heading word]	348,896	Advanced
6	caus*.mp. [mp = title, original title, abstract, name of substance word, subject heading word]	1,286,475	Advanced
7	risk*.mp. [mp = title, original title, abstract, name of substance word, subject heading word]	1,075,803	Advanced
8	6 or 4 or 7 or 5	2,901,862	Advanced
9	8 and 3	768	Advanced
10	The titles and abstracts of references from step 9 overviewed	222	Manual

## References

[1] Rose G Sick individuals and sick populations Int. J. Epidemiol 1985 14 32 38 3872850 10.1093/ije/14.1.32

[2] Wolfson MC Social proprioception: measurement, data, and information from a population health perspective Why Some People Are Healthy and Others Are Not? The Determinants of Health in Populations Evans RG Barer ML Marmor TR Aldine De Gruyter New York, NY, USA 1994 287 316

[3] Vanobbergen J Martens L Lesaffre E Bogaerts K Declerck D Assessing risk indicators for dental caries in the primary dentition Community Dent. Oral Epidemiol 2001 29 424 434 11784285 10.1034/j.1600-0528.2001.290603.x

[4] Steiner M Helfenstein U Marthaler TM Dental predictors of high caries increment in children J. Dent. Res 1992 71 1926 1933 1452896 10.1177/00220345920710121401

[5] van Palenstein Helderman WH van’T Hof MA van LC Prognosis of caries increment with past caries experience variables Caries Res 2001 35 186 192 11385198 10.1159/000047454

[6] Helderman WH Mulder J van’T Hof MA Truin GJ Validation of a Swiss method of caries prediction in Dutch children Community Dent. Oral Epidemiol 2001 29 341 345 11553106 10.1034/j.1600-0528.2001.290503.x

[7] Abernathy JR Graves RC Bohannan HM Stamm JW Greenberg BG Disney JA Development and application of a prediction model for dental caries Community Dent. Oral Epidemiol 1987 15 24 28 3467890 10.1111/j.1600-0528.1987.tb00475.x

[8] Petti S Hausen HW Caries prediction by multiple salivary mutans streptococcal counts in caries-free children with different levels of fluoride exposure, oral hygiene and sucrose intake Caries Res 2000 34 380 387 11014904 10.1159/000016612

[9] Raitio M Pienihakkinen K Scheinin A Multifactorial modeling for prediction of caries increment in adolescents Acta Odontol. Scand 1996 54 118 121 8739144 10.3109/00016359609006016

[10] Stamm JW Disney JA Graves RC Bohannan HM Abernathy JR The University of North Carolina Caries Risk Assessment Study. I: Rationale and content J. Public Health Dent 1988 48 225 232 3184028 10.1111/j.1752-7325.1988.tb03203.x

[11] Stewart PW Stamm JW Classification tree prediction models for dental caries from clinical, microbiological, and interview data J. Dent. Res 1991 70 1239 1251 1918574 10.1177/00220345910700090301

[12] Vanobbergen J Martens L Lesaffre E Bogaerts K Declerck D The value of a baseline caries risk assessment model in the primary dentition for the prediction of caries incidence in the permanent dentition Caries Res 2001 35 442 450 11799285 10.1159/000047488

[13] Benn DK Dankel DD Kostewicz SH Can low accuracy disease risk predictor models improve health care using decision support systems Proc AMIA Symp 1998 577 581 9929285 PMC2232092

[14] Disney JA Graves RC Stamm JW Bohannan HM Abernathy JR Zack DD The University of North Carolina Caries Risk Assessment study: further developments in caries risk prediction Community Dent. Oral Epidemiol 1992 20 64 75 1555390 10.1111/j.1600-0528.1992.tb00679.x

[15] Messer LB Assessing caries risk in children Aust. Dent. J 2000 45 10 16 10846266 10.1111/j.1834-7819.2000.tb00235.x

[16] Graves RC Disney JA Stamm JW Abernathy JR Bohannen HM Physical and environmental risk factors in dental caries Risk Assessment in Dentistry Bader JD Dental Ecology University of North Carolina Chapel Hill, NC, USA 1990 37 47

[17] Honkala E Nyyssonen V Kolmakow S Lammi S Factors predicting caries risk in children Scand. J. Dent. Res 1984 92 134 140 6585920 10.1111/j.1600-0722.1984.tb00869.x

[18] Hausen H Caries prediction—state of the art Community Dent. Oral Epidemiol 1997 25 87 96 9088697 10.1111/j.1600-0528.1997.tb00904.x

[19] Beck JD Disney JA Graves RC Stamm JW Kaste LM Bohannan HM University of North Carolina caries risk assessment study. Comparisons of high-risk prediction, any risk prediction, and any risk etiologic models Community Dent. Oral Epidemol 1992 20 313 321 10.1111/j.1600-0528.1992.tb00690.x 1464224

[20] Mattila ML Rautava P Paunio P Ojanlatva A Hyssala L Helenius H Sillanpaa M Caries experience and caries increments at 10 years of age Caries Res 2001 35 435 441 11799284 10.1159/000047487

[21] Broadbent JM Ayers KM Thomson WM Is attention-deficit hyperactivity disorder a risk factor for dental caries? A case-control study Caries Res 2004 38 29 33 14684974 10.1159/000073917

[22] Agrawal D Inappropriate interpretation of the odds ratio: oddly not that uncommon Pediatrics 2005 116 1612 1613 16322199 10.1542/peds.2005-2269

[23] Turunen S Nyyssonen V Vesala H Perspectives on poor dental health and its determinants Community Dent. Health 1992 10 49 55 8495393

[24] Baerum P Holst DRJ Dental Health in Trondelag 1983. Changes from 1973 to 1983 Directorate of Health Oslo, Norway 1985

[25] Unell L Soderfeldt B Halling A Birkhed D Explanatory models for clinically determined and symptom reported caries indicators in an adult population Acta Odontol. Scand 1999 57 132 143 10480278 10.1080/000163599428850

[26] Berset GP Eriksen HM Bjertness E Hansen BF Caries experience of 35-year-old Oslo residents and changes over a 20-year period Community Dent. Health 1996 13 238 244 9018890

[27] Gilbert GH Foerster U Dolan TA Duncan RP Ringelberg ML Twenty-four month coronal caries incidence: the role of dental care and race Caries Res 2000 34 367 79 11014903 10.1159/000016611

[28] Schwartz S Susser E Susser M A future for epidemiology? Annu. Rev. Public Health 1999 20 15 33 10352847 10.1146/annurev.publhealth.20.1.15

[29] Polit DF Hungler BP Nursing Research Principles and Methods J.B. Lippincott Company Philadelphia, NY, USA 1995

[30] Drake CW Hunt RJ Beck JD Koch GG Eighteen-month coronal caries incidence in North Carolina older adults J. Public Health Dent 1994 54 24 30 7909331 10.1111/j.1752-7325.1994.tb01175.x

[31] Eriksen HM Marques MD Bjertness E Moe B Dental caries determinants in an adult Portuguese population and a comparison with Norwegian adults Acta Odontol. Scand 1996 54 49 54 8669241 10.3109/00016359609003509

[32] Hallett KB O’Rourke PK Social and behavioural determinants of early childhood caries Aust. Dent. J 2003 48 27 33 14640154 10.1111/j.1834-7819.2003.tb00005.x

[33] Lawrence HP Hunt RJ Beck JD Three-year root caries incidence and risk modeling in older adults in North Carolina J. Public Health Dent 1995 55 69 78 7643330 10.1111/j.1752-7325.1995.tb02335.x

[34] Li Y Wang W Predicting caries in permanent teeth from caries in primary teeth: an eight-year cohort study J. Dent. Res 2002 81 561 566 12147748 10.1177/154405910208100812

[35] Campus G Solinas G Strohmenger L Cagetti MG Senna A Minelli L Majori S Montagna MT Reali D Castiglia P The Collaborating Study Group. National pathfinder survey on children’s oral health in Italy: pattern and severity of caries disease in 4-year-olds Caries Res 2009 43 155 162 19365120 10.1159/000211719

[36] Hansel PG Twetman S Bratthall D Evaluation of a computer program for caries risk assessment in schoolchildren Caries Res 2002 36 327 340 12399693 10.1159/000065963

[37] Seow WK Clifford H Battistutta D Morawska A Holcombe T Case-control study of early childhood caries in Australia Caries Res 2009 43 25 35 19136829 10.1159/000189704

[38] Tu YKKM Clerehugh V Gilthorpe MS Problems of correlations between explanatory variables in multiple regression analyses in the dental literature Br. Dent. J 2005 199 457 461 16215581 10.1038/sj.bdj.4812743

[39] Victora CG Huttly SR Fuchs SC Olinto MT The role of conceptual frameworks in epidemiological analysis: a hierarchical approach Int. J. Epidemiol 1997 26 224 227 9126524 10.1093/ije/26.1.224

[40] Hunt R Behavioral and sociodemographic risk factors for caries Risk Assessment in Dentistry Bader JD University of North Carolina Chapel Hill, NC, USA 1990 29 34

[41] Batchelor P Sheiham A The limitations of a ‘high-risk’ approach for the prevention of dental caries Community Dent. Oral Epidemiol 2002 30 302 312 12147172 10.1034/j.1600-0528.2002.00057.x

[42] Johnson NW Introduction: the nature of the caries process and the need for markers of risk Dental Caries Johnson NW Cambridge University Press Cambridge, UK 1991 1 12

[43] Risk Assessment in Dentistry Bader JD University of North Carolina Chapel Hill, NC, USA 1990

[44] Beck JD Kohout FJ Hunt RJ Heckert DA Root caries: physical, medical and psychosocial correlates in an elderly population Gerodontics 1986 32 42 47 3481708

[45] Locker D Leake JL Coronal and root decay experience in older adults in Ontario, Canada J. Public Health Dent 1993 53 158 164 8371194 10.1111/j.1752-7325.1993.tb02695.x

[46] Stamm JW Disney JA Beck JD Weintraub JA The University of North Carolina Caries Risk Assessment Study: final results and some alternative modelling approaches Cariology for the Nineties Bowen HW Tabak LA University of Rochester Press Rochester, NY, USA 1993 209 233

[47] Tubert-Jeannin S lardon JP Pham E Martin JL Factors affecting caries experience in French adolescents Community Dent. Oral Epidemiol 1994 22 30 35 8143439 10.1111/j.1600-0528.1994.tb01565.x

[48] Perera I Ekanayake L Factors influencing perception of oral health among adolescents in Sri Lanka Int. Dent. J 2008 58 349 355 19145796 10.1111/j.1875-595x.2008.tb00356.x

[49] Greenland S Gago-Dominguez CJE The value of risk-factor (“black box”) epidemiology Epidemiology 2004 15 529 535 15308951 10.1097/01.ede.0000134867.12896.23

[50] Karhausen LR Causation: the elusive grail of epidemiology Med. Health Care Philos 2000 3 59 67 11080970 10.1023/a:1009970730507

[51] Kay-Tee K Rose’s Strategy of Preventive Medicineupdated ed;Oxford University Press Oxford, UK 2008

[52] Porta M A Dictionary of Epidemiology5th ed Oxford University Press New York, NY, USA 2008

[53] Doyle YG Furey A Flowers J Sick Individuals and Sick Populations: 20 Years Later Eastern Region Public Health Observatory, Institute of Public Health Cambridge, UK 2005

[54] Weed DL Environmental epidemiology: basics and proof of cause-effect Toxicology 2002 181 399 403 12505342 10.1016/s0300-483x(02)00476-6

[55] Weed DL Theory and practice in epidemiology Ann. NY Acad. Sci 2001 954 52 62 11797865 10.1111/j.1749-6632.2001.tb02746.x

[56] Weed DL Precaution, Prevention, and Public Health Ethics J. Med. Philos 2004 29 313 332 15512975 10.1080/03605310490500527

[57] Donaldson AN Everitt B Newton T Steele J Sherriff M Bower E The effects of social class and dental attendance on oral health J. Dent. Res 2008 87 60 64 18096895 10.1177/154405910808700110

[58] Vanobbergen J Martens L Lesaffre E Bogaerts K Declerck D Assessing risk indicators for dental caries in the primary dentition Community Dent. Oral Epidemiol 2001 29 424 434 11784285 10.1034/j.1600-0528.2001.290603.x

[59] Rockhill B Theorizing about causes at the individual level while estimating effects at the population level: implications for prevention Epidemiology 2005 16 124 129 15613957 10.1097/01.ede.0000147111.46244.41

[60] Susser E Schwartz S Are social causes so different from all other causes? A comment on Sander Greenland Emerg. Themes Epidemiol 2005 24 2 4 10.1186/1742-7622-2-4 PMC1187907 15913455

[61] Susser M Susser E Choosing a future for epidemiology: from black box to Chinese boxes and eco-epidemiology Am. J. Public Health 1996 86 674 677 8629718 10.2105/ajph.86.5.674 PMC1380475

[62] Parascandola M Weed DL Causation in epidemiology J. Epidemiol. Community Health 2001 55 905 912 11707485 10.1136/jech.55.12.905 PMC1731812

[63] Mann CJ Observational research methods. Research design. II: cohort, cross sectional, and case-control studies Emerg. Med. J 2003 20 54 60 12533370 10.1136/emj.20.1.54 PMC1726024

[64] Barreto ML Epidemiologists and causation in an intricate world Emerg. Themes Epidemiol 2005 24 2 3 10.1186/1742-7622-2-3 PMC1180463 15913457

[65] Rose G The Strategy of Preventive Medicine Oxford University Press Oxford, UK 1993

[66] Maldonado GGS The causal-contrast study design Am. J. Epidemiol 2000 151 S39

[67] Earp JA Ennett ST Conceptual models for health education research and practice Health Educ. Res 1991 6 163 171 10148689 10.1093/her/6.2.163

[68] Phillips LR Causal modeling as a relevant approach to gerontological nursing J. Gerontol. Nurs 1990 16 20 24 2398227 10.3928/0098-9134-19900901-08

[69] Kar SB Berkanovic E Indicators of behaviour conducive to health promotion Measurement in Health Promotion and Protection Abelin T Brzezinski ZJ Carstairs VDL World Health Organisation Regional Office for Europe Copenhagen, Denmark 1987 267 293

[70] McHorney CA Health status assessment methods for adults: past accomplishments and future challenges Annu. Rev. Public Health 1999 20 309 335 10352861 10.1146/annurev.publhealth.20.1.309

[71] Greenland S Interpretation and choice of effect measures in epidemiologic analysis Am. J. Epidemiol 1987 125 761 768 3551588 10.1093/oxfordjournals.aje.a114593

[72] Evans RG Barer ML Marmor TR Why Are Some People Healthy and Others Not? The Determinants of Health of Populations Aldine De Gruyter New York, NY, USA 1994

[73] Altman DG Statistics in medical journals: some recent trends Statist. Med 2000 19 3275 3289 10.1002/1097-0258(20001215)19:23<3275::aid-sim626>3.0.co;2-m 11113959

[74] Cohen JT The cost of dichotomization Applied Psychological Measurement 1983 7 249 253

[75] Jacobsen BS Organising and displaying data Statistical Methods for Health Care Research Munro BH Lippincot Philadelphia, NY, USA 1997 3 29

[76] Aleksejuniene J Holst D Eriksen HM Patterns of dental caries and treatment experience in elderly Lithuanians Gerodontology 2000 17 77 86 11808058 10.1111/j.1741-2358.2000.00077.x

[77] Foxman B Challenges of epidemiology in the 21st century: comments from the leaders of several epidemiology associations Ann. Epidemiol 2005 15 1 4 15614967 10.1016/j.annepidem.2004.09.006

[78] Altman DG Practical Statistics for Medical Research Chapman and Hall London, UK 1993

[79] Lewis-Beck M Applied Regression SAGE Publications Beverly Hills, CA, USA 1980

[80] Koopman JS Lynch JW Individual causal models and population system models in epidemiology Am. J. Public Health 1999 89 1170 1174 10432901 10.2105/ajph.89.8.1170 PMC1508689

[81] Aleksejuniene J Holst D Sandvik L Prediction and explanation—two approaches for studying lifestyle in relation to oral health Trends in Lifestyle and Health Research Kinger L Nova Science Inc New York, NY, USA 2005

[82] Scheutz F Frydenberg M Matee MI Poulsen S The effect of choosing different units of analysis when estimating risk of presence of dental caries in the primary dentition Community Dent. Health 2003 20 27 33 12688601

[83] Dean K Integrating theory and methods in population health research Population Health Research: Linking Theory and Methods Dean K Sage Publications Thousand Oaks, CA, USA 1993 9 37

[84] Bokhout B Hofman FXLJ Prahl-Andersen BA ‘Sufficient cause’ model for dental caries J. Epidemiol. Biostat 2000 5 203 208 11051116

[85] Diez-Roux AV Multilevel analysis in public health research Annu. Rev. Public Health 2000 21 171 192 10884951 10.1146/annurev.publhealth.21.1.171

[86] Greenland S Brumback B An overview of relations among causal modelling methods Int. J. Epidemiol 2002 31 1030 1037 12435780 10.1093/ije/31.5.1030

[87] Byrne BM Structural Equation Models Structural Equation Modeling with EQS and EQS/Windows SAGE Publications Thousand Oaks, CA, USA 1994 3 22

[88] Bollen K Long J Testing Structural Equation Models Sage Publications Newbury Park, CA, USA 1993

[89] Rasbash J Steele F Browne WG Goldstein H A User’s Guide to MLwiN version 2.10 Centre for Multilevel Modelling, University of Bristol Bristol, UK 2009

[90] Newton JT Bower EJ The social determinants of oral health: new approaches to conceptualizing and researching complex causal networks Community Dent. Oral Epidemiol 2005 33 25 34 15642044 10.1111/j.1600-0528.2004.00190.x

[91] Greenland S Morgenstern H Confounding in health research Annu. Rev. Public Health 2001 22 189 212 11274518 10.1146/annurev.publhealth.22.1.189

[92] Oh HS Seo WS Development of a structural equation model for causal relationships among arteriosclerosis risk factors Public Health Nurs 2001 18 409 417 11737809 10.1046/j.1525-1446.2001.00409.x

[93] Litt MD Reisine S Tinanoff N Multidimensional causal model of dental caries development in low-income preschool children Public Health Rep 1995 110 607 617 7480616 PMC1381639

[94] Aleksejuniene J Holst D Eriksen HM Gjermo P Psychosocial stress, lifestyle and periodontal health J. Clin. Periodontol 2002 29 326 335 11966930 10.1034/j.1600-051x.2002.290408.x

[95] Aleksejuniene J Holst D Grytten JI Eriksen HM Causal patterns of dental health in populations. An empirical approach Caries Res 2002 36 233 240 12218271 10.1159/000063923

[96] Fliege H Rose M Arck P Walter OB Kocalevent RD Weber C Klapp BF The Perceived Stress Questionnaire (PSQ) reconsidered: validation and reference values from different clinical and healthy adult samples Psychosom. Med 2005 67 78 88 15673628 10.1097/01.psy.0000151491.80178.78

[97] Donaldson AN Everitt B Newton T Steele J Sherriff M Bower E The effects of social class and dental attendance on oral health J. Dent. Res 2008 87 60 64 18096895 10.1177/154405910808700110

[98] Bower E Gulliford M Steele J Newton T Area deprivation and oral health in Scottish adults: a multilevel study Community Dent. Oral Epidemiol 2007 35 118 129 17331153 10.1111/j.1600-0528.2007.00308.x

